# Monocular visual loss due to herniation of gyrus rectus: A case report with review of literature

**DOI:** 10.4103/0974-620X.60027

**Published:** 2010

**Authors:** K. Sharma, A. K. Srivastava, V. Kanaujia, S. Jaiswal, A. K. Jaiswal

**Affiliations:** Department of Neurosurgery and Pathology, Sanjay Gandhi Postgraduate Institute of Medical Sciences, Lucknow, India

**Keywords:** Gyrus rectus, anterior visual pathway, suprasellar cistern

## Abstract

The authors present an unusual case of a polar mass in the frontal lobe of the brain, causing acute monocular visual loss in a 50-year-old woman with history of breast carcinoma treated with surgery, radiation and chemotherapy. Neuroimaging demonstrated herniation of the gyrus rectus into the suprasellar cistern resulting in compression of the anterior visual pathway.

## Introduction

Frontal lobe tumors often produce impairment in cognitive and motor functions of the patient. They can produce visual loss either from chronic papillaedema or by direct compression of the optic nerve and chiasm. The latter is often caused by tumors located in the posterior part of the inferior surface of the frontal lobe.[1] We report an unusual case of a frontal lobe mass located near the pole (anterior end) causing acute monocular visual loss. This occurred through compression of the anterior visual pathway by the gyrus rectus, which had herniated into the suprasellar cistern. The gyrus rectus, an anatomic structure located at the very middle in the anterior cranial fossa, is in close vicinity of the intracranial part of optic nerve and anterior part of the chiasma.

## Case Report

**Figure 1:**
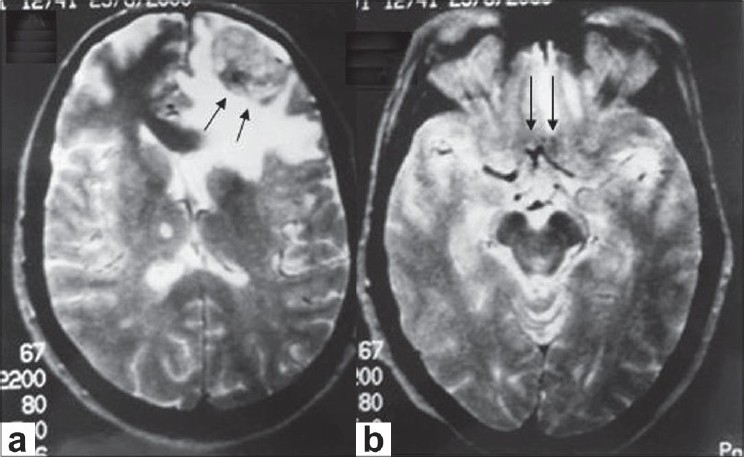
Magnetic resonance imaging brain axial section T2-weighted images showing (a) a rounded mass (arrow) in the polar region of the left frontal lobe; (b) herniation of the left gyrus rectus (arrow) resulting in obliteration of the suprasellar cistern

A 50-year-old nondiabetic and nonhypertensive woman complained of painless, progressive loss of vision in her left eye (OS) of one week duration. She did not have any history suggestive of raised intracranial pressure. There was no history of trauma. One year back she had undergone radical mastectomy for breast carcinoma followed by radiotherapy and chemotherapy. Ophthalmic examination OS showed visual acuity of no light perception. There was an afferent pupillary defect. Examination of the ocular motility and fundus was unremarkable. Examination of the right eye including visual field by confontration method showed no abnormalities. Systemic examination revealed ascites and hepatomegaly. Magnetic Resonance Imaging (MRI) of the brain showed a rounded mass in the polar region of the left frontal lobe with significant edema in the surrounding region [Figure 1a]. The left gyrus rectus side was herniating into the suprasellar cistern causing its obliteration [Figure 1b]. A radiological diagnosis of metastasis was made. Metastatic work up was planned including chest X-ray, ascitic fluid examination, liver biopsy and bone scan, but the patient died three days after presentation due to myocardial infarction. Patient’s relatives refused for autopsy.

## Discussion

Our patient was a known case of breast carcinoma with metastasis to the frontal lobe, liver and peritoneum. The frontal mass was located near the anterior end of the frontal lobe and did not cause any neurological deficit. MRI of the brain showed considerable edema of the medial aspect of the frontal lobe and herniation of right gyrus rectus in the suprasellar cistern causing its obliteration. The extensive edema around the mass had apparently extended to the gyrus rectus causing it to herniate into the suprasellar cistern with compression of intracranial part of optic nerve and anterior chiasma. Clinically, the patient demonstrated only features of optic nerve compression. Careful examination of the visual field OD did not show any defect despite apparent anterior optic chiasmal compression.[2] Herniation of the gyrus rectus compressing the optic chiasma has been reported by Walsh and Gass[2] but they did not confirm the clinical features with radiological studies. Lindenberg[3] in an autopsy discussed the possibility of herniation of the gyrus rectus causing visual loss. Klingler *et al*.[4] reported a case of meningioma of the posterior falx cerebri who had sign and symptoms of raised intra cranial pressure and bilateral visual loss due to herniation of the gyrus rectus into the spurasellar cistern with resultant compression of the optic chiasma. They demonstrated on computerized tomography (CT) brain the distortion of the pentagonal configuration of the suprasellar cistern by the herniating gyrus rectus. Follow-up CT showed a normal appearing suprasellar cistern.

Usually, the prognosis in cases of brain metastasis following breast carcinoma is poor. In our case, the radiological findings of significant edema around the lesion and the previous history of surgery, radiotherapy and chemotherapy for breast carcinoma led to a provisional diagnosis of brain metastasis. In many cases, patients are not forthcoming about previous history of malignancy leading to delays in the diagnosis. Our case emphasizes the fact that visual loss can result from a tumor of the frontal lobe located some distance away from the anterior visual system, and the importance of obtaining detailed clinical information including history of previous malignancies in arriving at the right diagnosis and for timely management.
